# Lumenless and Stylet-Driven Leads for Left Bundle Branch Area Pacing: Materials, Techniques, Benefits, and Trade-Offs of the Two Approaches

**DOI:** 10.3390/jcm13164758

**Published:** 2024-08-13

**Authors:** Simone Taddeucci, Gianluca Mirizzi, Amato Santoro

**Affiliations:** 1 Division of Biotechnology, University of Siena, Viale Bracci 1, 53100 Siena, Italy; s.taddeucci1@student.unisi.it; 2 Division of Cardiology, Tuscany Foundation ‘Gabriele Monasterio’, Via Moruzzi, 1, 56100 Pisa, Italy; gianluca.mirizzi@ftgm.it; 3 Division of Interventional Cardiology, Azienda Ospedaliera Universitaria Senese, Viale Bracci 1, 53100 Siena, Italy

**Keywords:** left bundle branch area pacing, lumenless leads, stylet-driven leads, pacemaker implantation

## Abstract

Left bundle branch area pacing (LBBPa) is an innovative technique for physiological pacing. Compared with His bundle pacing, LBBPa provides better pacing thresholds, lower rates of macrodislodgment, and a reliable strategy for cardiac resynchronization. LBBPa traditionally employs lumenless leads (LLL), which are characterized by small lead bodies and a fixed helix design. These features guarantee stability, avoid helix retraction, and facilitate easier septal penetration, all contributing to an advantageous learning curve. On the other hand, stylet-driven pacing leads (SDL) have shown comparable success rates related to lumenless pacing leads, although they carry risks of helix retraction and lead fracture. SDL have been increasingly employed with favorable results, as they provide good maneuverability and support during implantation with continuous monitoring of ECG-paced morphology. Different manufacturers are offering a variety of SDL, and new dedicated tools are being developed to simplify lead implantation. In this review, we examine the procedural techniques, advantages, and limitations of the most commonly used pacing leads and tools for LBBPa, and we summarize the complications associated with both lumenless leads (LLL) and stylet-driven leads (SDL).

## 1. Introduction

Left bundle branch pacing area (LBBPa) is an emerging technique that enables resynchronization of the left ventricle through the capture of left bundle branch (LBB) arborizations. The objective of this technique is to capture the conduction system along the LBB or its fascicles with conventional or adapted pacing leads through dedicated delivery sheaths. The His bundle pacing (HBP) technique was initially introduced as a conduction system pacing (CSP) technique, but its advantages are counterbalanced by high thresholds, sensing issues, lower capacity of resynchronization in case of bundle branch block, and high displacement rates compared with other pacing techniques. Backup pacing leads are often required in HBP for safety concerns. LBBPa usually guarantees better thresholds and lower rates of macrodislodgment compared with HBP [1]. The LBBPa experience is primarily derived from lumenless leads (LLL), in particular the SelectSecure™ 3830 (Medtronic, Minneapolis, MN, USA), but stylet-driven leads (SDL) have been increasingly utilized. Short- and mid-term observational studies have demonstrated higher acute implant success rates with LLL, but comparable proportions of LBBPa capture and complication rates [2]. Sritharan A. et al. reported comparable success rates after a 50-patient learning curve [3]. The mostly used SDL are the Solia S pacing lead (Biotronik, SE & Co., Berlin, Germany), the Ingevity pacing lead (Boston Scientific Inc., Marlborough, MA, USA), and the Tendril 2088TC pacing lead (Abbott, Inc., Chicago, IL, USA). While SDL have a long track record of reliability for traditional pacing, CSP portends new challenges and complications such as early lead fracture, tricuspid regurgitation, macro lead dislodgment, and microdislodgment. Acute complications of LBBPa include septal perforation during the procedure [4]. In this paper, we summarize the procedural techniques and possible complications for both LLL and SDL implantation, highlighting the primary advantages and disadvantages of these leads in the short- and mid-term following pacemaker implantation.

## 2. LBBP Lead Placement Technique

**Figure 1:**
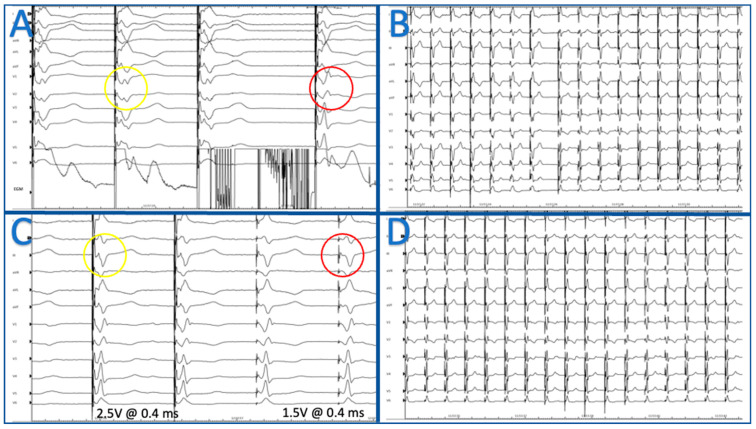
Panel (**A**): Septal mapping and W sign in V1 during pacing at 100 mm/s (yellow circle); transition during septal penetration, from W sign to rsR’ on V1 (red circle). Panel (**B**): Same ECG reproduced at 25 mm/s. Panel (**C**): Non selective LBBPa at 2.5 V × 0.4 ms without isoelectric line (yellow circle); selective capture of LBBPa at lower output (red circle). Panel (**D**): LBBPa pacing ECG at 25 mm/s.

The pacing lead is initially advanced through the sheath, and the unipolar signal from the tip of the lead should be displayed. Optimal filtering for high-frequency signals (30–500 Hz) should be used to identify conduction system signals. Optimal delivery sheaths should allow for the easy identification of His potential. In the absence of a His signal, tricuspid valve potentials (local EGMs both from the atrium and from the ventricle separated by an isoelectric interval) can substitute this information, and contrast injection can confirm the delivery position. After obtaining a His electrogram under unipolar configuration, the whole system is advanced 2–3 cm in right anterior oblique (RAO) projection along the LBB area. RAO projection allows the identification of antero-posterior anatomy and appropriate advancement to the LBBPa. A pure fluoroscopic approach is also possible, dividing the RV silhouette in RAO into nine segments from base to apex, advancing the guiding lead while aiming at the central segment of the basal and medial parts of the RV [5]. Unwanted coronary sinus cannulation frequently occurs both with double-curve fixed delivery or deflectable devices and can be confirmed in left anterior oblique projection. In contrast to the His bundle and the right bundle branch, the LBB demonstrates a notable fan-like dispersion of fibers into the septal myocardium, exhibiting less stringent anatomical demarcations. This dispersion usually facilitates the engagement of the conduction system but can be a determinant of failure to achieve selective LBBPa due to wide interindividual variability. Subsequently, in LAO, it is possible to identify the correct site by observing the septal curve of the sheath pointing to the septum between 2 and 3 o’clock. Contrast injection from the delivery can confirm the septal position and perpendicular lead orientation in LAO projection. This is a crucial aspect of lead positioning, particularly when using the LLL lead. Due to its “floppy” nature, without a perpendicular delivery position and tight adherence to the ventricular septum, the LLL can bend and fail to penetrate the septum perpendicularly, which is necessary for achieving conduction tissue pacing stimulation. On the other hand, while using the SDL, strict adherence to the septum is not necessary for septal penetration, as the SDL is more supportive. However, in the case of excellent contact and adequate alignment with the septum, LLL allows deep penetration and adequate LBBPa due to the electrode structure and screw dimensions as described below (Medtronic advantages paragraph). Unipolar pacing at this site should yield a “W” pattern in V1 with discordant or negative concordant paced QRS in leads II and III (Figure 1A,B). In the case of QRS negative concordance, the lead may point inferiorly to the region of the posterior fascicle, whether a positive concordance suggests the anterior fascicle as a landing site. Initial clockwise screws can be applied up to 10 turns; an average of 8–12 turns have been reported with LLL but can widely vary according to delivery sheath position, septal thickness, and myocardial pathological conditions [6]. The specific characteristics and implantation techniques for the SDL lead are discussed in the subsequent paragraphs. During lead screwing, the delivery must be firmly in contact with the myocardium and perpendicular to the septum; in the case of large tracts of lead outside the sheath, torque forces may spread over the body lead, causing supercoiling without effective deep penetration. As mentioned above, repositioning the delivery sheath can favor effective rotations. The paced QRS precordial leads should have an early–mid-transition with a positive QRS complex in V6 for LBBPa, which is fundamental for LBBB capture, and typically shows an Rs pattern or RS in the case of more apical positions [7]. A subsequent approach based on a stepwise approach, as reported in the EHRA clinical consensus statement on CSP implantation:

- QRS transition morphology during pacing thresholds;
- R wave peak time in V6 (V6RWPT) < 75 ms in case of native narrow QRS or RBBB; <80 ms in case of LBBB or asystole, or intra ventricular conduction delay with RBBB and fascicular block; in patients with severely dilated LV or conduction system disease, the accepted cutoff may be <90 ms;
- Measurement of V6–V1 inter-peak interval > 44 ms;
- QRS transition to selective LBBPa during programmed stimulation is recom-mended for assessing LBBPa capture, as showed in Figure 1C,D [8].

In addition, frequent checks of impedance, unipolar unfiltered current of injury and paced QRS can determine the criteria of LBB capture and reduce the risk of septal perforation (see complications paragraph). 

Selective (S-LBBP) and non-selective LBBPa (NS-LBBP) can be identified at the time of lead placement: Unipolar pacing should be performed as the lowest output to achieve selective capture S-capture (usually 0.5–2.0 V × 2 ms) with increasing output to achieve NS capture. 

S-LBB capture is suggested by the following intracardiac and surface criteria:

- absence of local ventricular EGM capture into stimulus artifact with discrete isoe-lectric components on the paced bipole pair and immediately adjacent bipole pairs (2-mm interspacing) at EGM.
- presence of an isoelectric interval between the pacing spike and ECG QRS complex.
- the pacing spike–QRS interval closely approximates the left Purkinje potential to QRS interval.

NS-LBB capture is suggested by the following intracardiac and surface criteria:

- direct capture of the local ventricular EGM into the pacing stimulus artifact with recruitment of ventricular components on the immediately adjacent bipole pairs (2-mm interspacing).
- absence of isoelectric interval between the pacing spike and ECG QRS complex [8].

S-LBBP predominantly yields a wide QRS duration as a result of delayed RBB syn-chronization, whereas NS-LBBP results in shorter QRS. In most patients, achievement of narrower QRS during LBBPa can be produced by fusion with intrinsic right bundle ac-tivation and NS LBBPa [8,9]. 

## 3. Lumenless Lead

### 3.1. Medtronic

**Figure 2:**
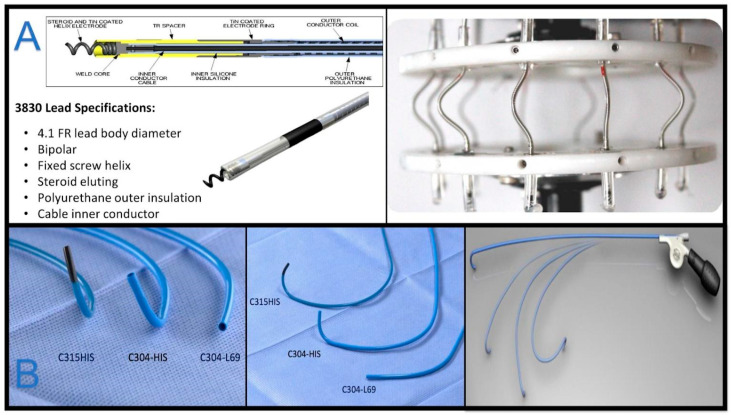
Medtronic materials are shown. In Panel (**A**), SelectSecure™ 3830 features are listed. In vitro and in vivo analysis assessed the lead’s durability under extreme bending conditions, predicting a 10-year fracture rate of 0.02% [6]. The lower Panel (**B**) shows Medtronic C315 fixed curve delivery sheaths and the deflectable C304 delivery sheath.

The implantation technique for LBBPa with LLL has been extensively described by Huang et al. [10]. The SelectSecure™ MRI SureScan™ 3830 (Medtronic) LLL bipolar pacing lead, a 4.1 F LLL, features a fixed helix and isodiametric tip with excellent tensile strength for LBBPa. SelectSecure™ 3830 (Medtronic) is the smallest commercialized lead for LBBPa. Standard lengths available are 59, 69, and 74 cm, with an additional non-MRI length of 49 cm for specific applications. The materials used in the construction of the lead include a polyurethane outer insulator, with silicone and ethylene tetrafluoroethylene (ETFE) as inner insulators. The conductor is made of MP35N, a durable and corrosion-resistant alloy. Both the helix and ring electrodes are coated with titanium nitride on a platinum alloy base, enhancing conductivity and biocompatibility. Additionally, the lead incorporates the steroid beclomethasone dipropionate to minimize inflammation at the implantation site. Dedicated delivery sheaths are required to give support and stability for the location of the distal His electrogram, subsequent location of the LBB area, and screwing. Delivery sheaths can be distinguished into 3D double-curve fixed sheaths (C315 His, Medtronic Inc, Minneapolis, MN, USA) and deflectable curve sheaths (C304, Medtronic Inc, Minneapolis, MN, USA). Both C315 and C304 delivery sheaths present a 3D shape with a primary and a secondary curve (see Figure 2). C304 presents with a larger inner lumen of 5.7 F compared with C315 (5.4 F), which is also furnished with a hydrophilic coating. Outer diameters vary from 8.4 F in C304 to 7 F in C315. The secondary curve is fundamental to reaching the His region, as it is also called the “his shaped curve” and is always preshaped. According to our experience, the C315 secondary curve is more angulated and guarantees easier access to the His bundle region, but the C304 deflectable sheath can be an additional option for difficult anatomies.

### 3.2. Advantages

According to its dimensions and features, SelectSecure™ 3830 (Medtronic, MN, USA) is linked to minimal myocardial injury, improved long-term outcomes, and reduced helix damage and procedural complications. Although SDL are being explored, LLL remain the preferred choice globally for LBBPa due to their extensive experience [2] and their well-documented implantation technique [10]. This lead uniquely features a helix that maintains an isodiametric configuration with the lead body, facilitating smoother septal penetration compared with other leads, and its exposed helix design avoids helix retraction. The 1.8 mm helix is tightly connected to the coil, which guarantees further strength to the system, possibly reducing the risk of helix rupture [11]. A recent in vitro and in vivo analysis assessed the lead’s durability under extreme bending conditions, predicting a 10-year fracture rate of 0.02%. The results indicated that LBBPa implants require more rotations and result in unique lead bending, but accelerated testing showed no excess conductor fractures [12]. LLL use was first developed for unconventional pacing sites, particularly in congenital heart diseases, but thanks to its versatile pacing site, it has received CE approval for CSP and is the most commonly used lead for this purpose.

### 3.3. Limitations

Lead extraction in the LBBPa area with LLL is a main concern, as it does not allow for the use of a locking stylet. The high torquability of the lead with a lumenless design and high tensile force may cause technical difficulties during the extraction procedure, which could require the use of lead extenders and compression ties. While a non retractable helix with an isodiametric shape is an advantage during lead positioning in LBBPa, its solid shape can cause perforation and avulsion of larger pieces of myocardial tissues adhered to the tip during lead extraction, possibly causing permanent conduction system damage. Initial experiences have been reported with lead extractions, and no major complications were reported in a small cohort of patients [13,14,15], but further investigations are required. Despite substantial experience with this pacing lead, compared with SDL, LLL may lack support, stability, and steerability during transseptal screwing for LBBPa.

## 4. Stylet-Driven Leads

SDL have been the traditional choice for right ventricular apex pacing. These leads are stiffened with a stylet during implantation along their lumen. The first two cases of LBBPa were successfully reported by Zanon et al. [16] with a Selectra 3D delivery sheath (Biotronik) and a Solia S 60 (Biotronik) pacing lead. After 4 years of worldwide experience, the LBBPa success rate with SDL was reported to be 96.1% [3]. Different manufacturers are investing in this approach with dedicated solutions and common basic principles but slightly different workflows in the handling of the leads and materials. In the next paragraphs, we will try to summarize the main procedural aspects for achieving LBBPa with currently available SDL—Abbott, Biotronik, and Boston Scientific—and provide specific delivery leads to approach LBBPa using their commercially available leads to perform LBBPa.

### 4.1. Advantages

LBBPa with SDL offers several advantages: SDL sheaths are slightly more rigid and transfer torque without kinking, and the presence of stylets with different degrees of stiffness adds to maneuverability and offers more support for septal penetration. Most importantly, continuous monitoring of ECG morphology during unipolar pacing, connecting the crocodile clamp to the stylet, allows for lead penetration to be precisely gauged while keeping maneuverability unaltered. The use of SDL in LBBPa has demonstrated a low lead revision and dislodgment rate similar to or lower than traditional leads, highlighting the method’s reliability [17]. However, some challenges, such as the risk of entanglement and the fragility of the extendable helix, require attention during the procedure. Overall, LBBPa with SDL proves to be a safe and feasible technique, but future lead development should aim to combine the advantageous features of both SDL and traditional leads to further enhance implant success and overall safety.

### 4.2. Limitations

Clockwise rotations of the outer lead body can cause helix retraction, and it can be avoided by specific actions, such as pretensioning the inner coil, ensuring better lead progression in the septal region, and continuous impedance check. Specific tools to lock the helix during lead penetration, designed for some leads (Biotronik, Abbott), reduce this risk. Further challenges are represented by the risk of entanglement and the fragility of the extendable helix, which can lead to helix deformation or fracture. During lead placement, the delivery sheath must be perpendicular to the septum. SDL, due to their supportive nature, may allow for non-perpendicular septal screw insertion without strict adherence of the delivery sheath to the septum, but selective LBBPa pacing is rarely achieved. Several attempts to screw the lead into the septum with incorrect angulations, followed by subsequent retractions of the helix, may lead to helix damage or fracture.

The risk of lead fracture is a major concern during LBBPa due to the presence of a fulcrum point and high friction. Observational studies have shown higher early lead fracture rates in SDL compared with LLL [18], but numbers are still very low, and long-term consideration cannot be made; furthermore, proper handling of the leads and maintaining proper alignment of the body and the distal end of it seems to provide protective effects. Various manufacturers sell different pacing lead lengths and a wide variety of delivery sheaths. Depending on the relative length of the assembled lead, the available space for delivery slitters can be constrained by factors such as the combined length of the lead with an IS1 pin and the delivery sheath. However, this problem can be easily overcome by cutting the lead’s sleeve before slittering, gaining additional space. The sleeve can be then repositioned for lead fixation. A significant consideration is related to stylet insertion: the stylet must be kept fully inserted during rotation maneuvers. Even a small retraction of the stylet prevents the advancement of the electrode in the septum. A “locking system” currently not available for any company, could help overcome this issue.

### 4.3. Solia S (Biotronik)

**Figure 3:**
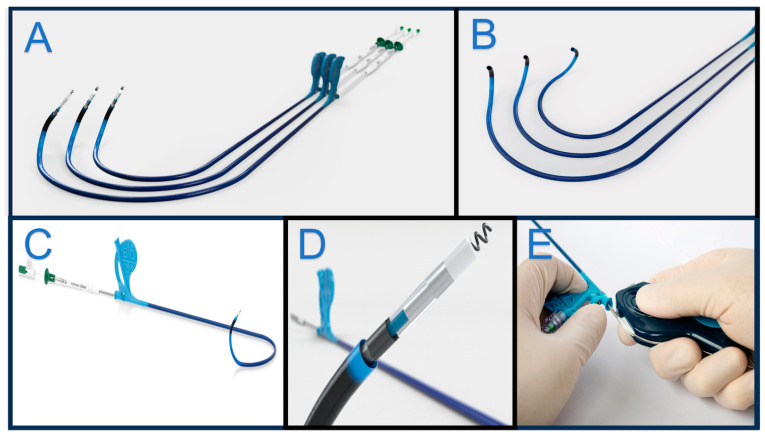
Biotronik delivery sheaths are shown in Panel (**A**,**B**) with three different 3D-shaped curves to accommodate different anatomies. Panel (**C**,**D**) show an assembled delivery with a stylet-driven Solia S lead. The green locking tool allows an easier helix deployment into the septum. Panel (**E**) shows Biotronik slitter over the lead.

Solia S pacing leads (Biotronik, Berlin, Germany) are the most popular SDL for LBBPa. Solia leads are bipolar pacing leads with a coaxial structure and silicone outer isolation commercialized in 2009. Preimplantation helix extension should be performed to pretension the inner coil and avoid helix retraction during manual clockwise rotation. SDL require delivery systems to reach the septal position, and the manufactured Selectra 3D delivery sheaths are the most used Biotronik Delivery systems (see Figure 3). Their design allows them to reach the His Bundle or LBB area, and they come in different lengths and shapes. Selectra 3D has three fixed curves and three possible lengths and is released with a dilatator for insertion, and the lumen for contrast media administration has an internal valve. They have a tungsten radiopaque tip and feature an internal hydrophilic coating. During lead preparation, the fixation tool should be attached to the connector pin and rotated to extend the helix fully by clockwise rotations. Further rotations of the fixation tool allow it to lock in an extended position. In the case of conventional anatomies, it is possible to advance the delivery sheath with the Solia S lead in advance without using the dilatator. This applies to all types of delivery sheaths. However, in instances of complex anatomical structures, advancing the delivery system over a wire using a dilatator may provide an effective solution.

During navigation with the stylet fully inserted, a unipolar signal with the cathode on the tip of the lead can identify a His signal. The whole system is advanced in LAO projection, and the paced QRS morphology (W sign) can suggest the right area to target for LBBPa. Using Solia S leads, the helix can be advanced during navigation into the sheath and before deployment; this technique may guarantee a more appropriate impedance starting value. If the helix was previously advanced, the lead can be screwed through clockwise rotation. During these maneuvers, the crocodile pinches should be connected to the stylet and clockwise rotation performed during unipolar pacing (tip-skin). Endocavitary electrograms should be displayed both filtered (30–500 Hz) or unfiltered (0.5–500 Hz). Unipolar unfiltered signals can help in identifying the COI, which progressively raises during septal penetration but falls in the case of perforation. During positioning, it is advisable to keep a firm position of the delivery to avoid lead stress at the fulcrum (the point of the lead between the tip of the delivery and the myocardium), which is the main position of lead fracture. Specific devices such as Amvia DR-SR-HF, Biotronik, and leads for stimulating the left branch have been manufactured and have received CE approval for physiologic stimulation.

### 4.4. Tendril™ STS 2088TC (Abbott)

The SDL from Abbott is a 5.8 Fr with a 2 mm extended helix. The outer isolation with Optim^®^ material, a silicone and silicone rubber copolymer, offers long-term resistance to abrasion. In LBBP, lead abrasions, friction, and risk of fracture are major concerns, and high-resistance materials can guarantee better results.

Abbott offers various delivery systems, such as the fixed curve CPS Locator^TM^ 3D leads family, released with five dimensions to accommodate various anatomies (small, medium, large, extra long medium, extra long large). In all cases, the outer diameter is 9 F, while the inner diameter is 7 F. A new CPS Direct™ Universal 3D delivery was released in 2023 and is a 3D shape 8 F (outer diameter 10 F) valved Abbott delivery system with a radiopaque distal tip marker, made in PEBAX™ with a decreasing durometry with lower stiffness at its extremity. During navigation with the stylet fully inserted, a unipolar signal with the cathode on the tip of the lead can identify the His signal. The whole system is advanced in LAO projection, and the paced QRS morphology (W sign) can suggest the right area to target for LBBPa (as described below). Using a Tendril lead, the helix should be advanced after navigation into the ventricular septum. Tendril lead implantation can be obtained through standard clockwise rotation of the connector pin only (up to 20–30 turns), as the helix mechanism is protected from overturning. As the helix grips into the myocardium, it advances with the lead body, and contrast injection through a dedicated external valve can confirm deep septal penetration.

**Figure 4:**
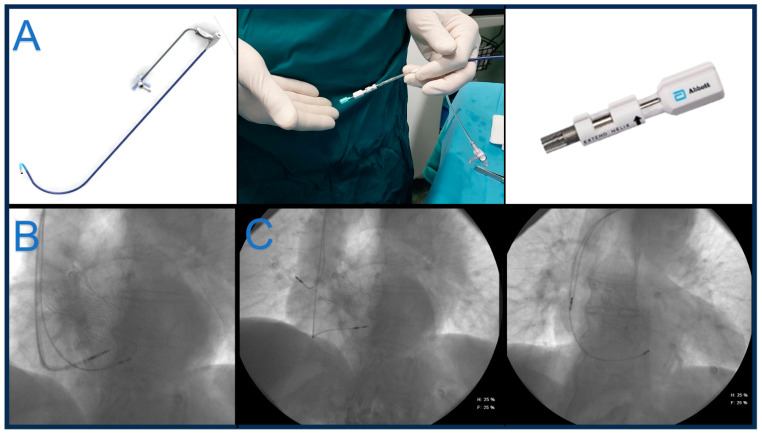
Panel (**A**). Abbott delivery and helix locking tool are shown. Panel (**B**): Tendril™ (Abbott) helix deployment in LAO projection. In this case, RA lead can be temporarily placed in RV septum as a backup lead during LBBPa lead positioning. Panel (**C**): Final position of the leads in LAO projection (**left** panel) and Ap view (**right** panel).

The new helix locking tool (Figure 4) allows for a new implantation technique, as it blocks the inner coil over the outer coil in IS-1 lead connectors and allows manual rotation and helix extension. The helix locking tool allows a safe outer body rotation and avoids helix retraction by co-rotating the outer and inner coils. The operator should be able to cease torquing and perceive eventual lead rotation, but while rotating the lead body, no counter torque should be perceived. Counter torques can be a sign of septal penetration failure (e.g., entanglement). Pinch tools can be applied to obtain electrical parameters along the locking tool dedicated spaces. It is compatible with St. Jude Medical patient cable models 4051A, 4051L, and 401748. The number of rotations that should be applied is not standardizable and should be individualized based on surface ECG findings and electrical parameters, as normal and pathological anatomical variants can determine variable thickness and stiffness. It is advisable to use Tendril 69 cm for LBBPa; in case the 58 cm is implanted, the sleeve can be removed to improve the delivery cut system, and longitudinal cuts allow easy repositioning of the sleeve.

### 4.5. Ingevity + (Boston Scientific)

**Figure 5:**
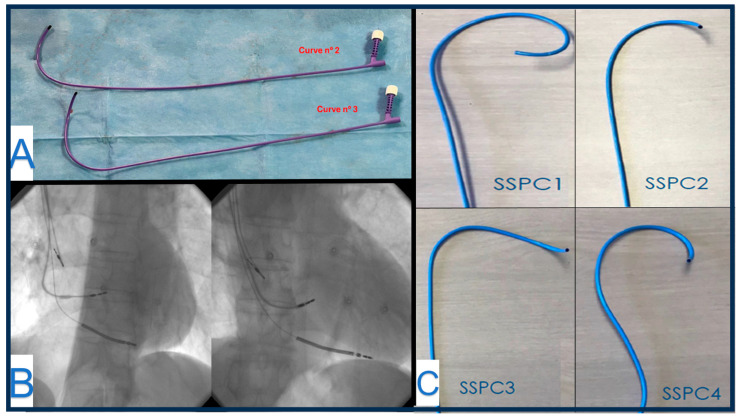
Panel (**A**): Boston Scientific offers three different 3D fixed curved delivery sheaths. SSPC3 Extended Hook Curves 2 and 3 are the most used. Panel (**B**): In panel B, an AP projection is shown on the left. Deep septal penetration is achieved by outer body torquing. On the right is an RAO projection of the same patient. Panel (**C**): Different curves from Site Selective Pacing Catheter (SSPC family). SSPC1 is a “C-shape” designed for RA septal locations. SSPC2: “Multipurpose” designed for RA and RV septal locations. SSPC3 is the “Extended hook” thought for septal locations like LBBPa. SSPC4 was designed for right-sided venous access to RA septal locations.

**Figure 6:**
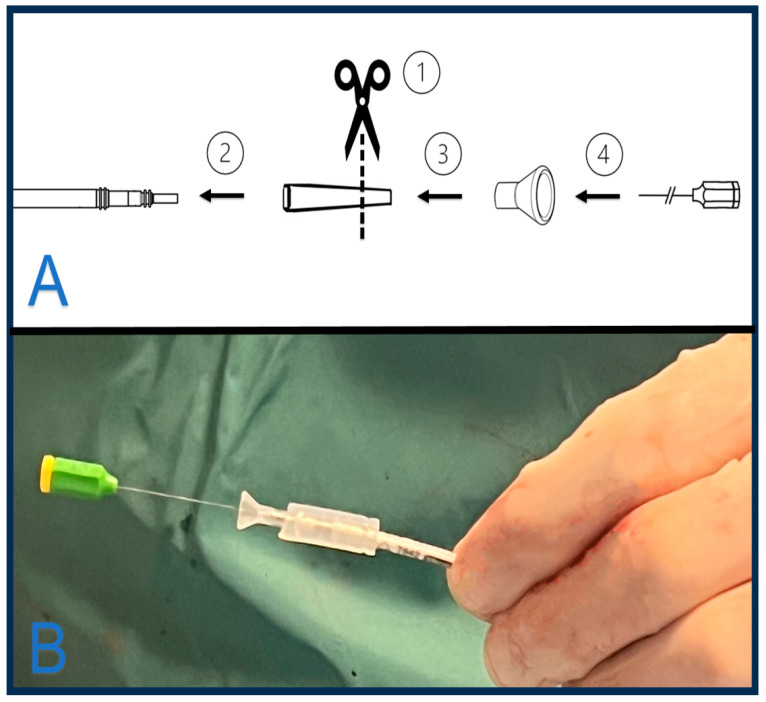
Panel (**A**) shows a customized locking tool for the Ingevity + catheter lead. Step 1: Cut the lead cap at ⅔ of the longer tail, as shown in the figure. Step 2: Insert it into the lead. Step 3: The insert tool is pushed under the cut tail of the cap and over the IS1 tip. The customized sleeve locks the inner coil and prevents helix retraction. Step 4: Insert the desired stylet. Panel (**B**) shows an assembled lead with the customized locking tool.

Ingevity Plus is a 6F stylet-driven lead high impedance design, with a co-radial trifilar inner coil of MP35N™ and a single-filar outer coil of MP35N (SPS Technologies, Inc., Santa Ana, CA, USA) with a silver core, that compared with the previous Ingevity leads, improves the torque transmission in the inner coil. Better efficiency on helix deployment and helix retraction are fundamental for LBBPa, as outer body rotation may cause helix retraction, and further clockwise torques of the pin must be applied to expose the helix in advance. Boston Scientific offers a variety of delivery sheaths into the so-called Site Selective Pacing Catheter (SSPC family), where SSPC 2 and 3 Extended Hook are the most used for LBBPa purposes (Figure 5). Three fixed curves can accommodate different anatomies. Curves are numbered from n°1, the smallest, to n°3, the larger. Two different implantation techniques can be used: In the first case, a first helix deployment allows a septal penetration with conventional clockwise rotation with the pinch tool. During screwing, the stylet guarantees more support and should be fully inserted into the tip. After the first 10–15 clockwise rotation, the helix is fully extended and can be confirmed in LAO projection. Subsequent advancement is obtained by torquing the body of the lead. After the helix penetration in the RV septum, the lead body should penetrate to obtain LBBPa. While delivering clockwise rotation to the outer body of the Ingevity+ lead, wider delivery sheaths like this tend to follow the torque, especially with larger n°3 sheaths, resulting in a loss of angulation with the septum. In this case, a slight 20–30° counter clock rotation of the delivery guide catheter over the pacing lead can restore the angulation. The delivery sheath position should be continuously assessed during lead screwing in 20–30° LAO projection with iodine contrast administration to maintain the perpendicularity and the vicinity of the delivery catheter to the septum. The delivery system SSPC 2 is commonly used as the first choice in most common anatomies because of its optimal angle, which ensures a good perpendicular contact of the lead with the RV septum. A second technique was described by De Pooter J et al. [17] and allows for the building of a customized locking tool to fix the inner coil and avoid helix retraction during body lead manual rotations. A silicone-cut lead cap is applied as a bridge between the stylet guide tool and the outer body of the lead (Figure 6). The stylet guide tool is forced under the customized lead cap and locks the inner coil during outer body manual rotations. This technique avoids helix retraction during septal penetration.

## 5. Potential Complications in LBBPa

- Septal perforation. Septal perforation is a common complication and is reported in up to 10.2% of LLL and 11.6% of SDL. The procedural outcome and follow-up of SDL compared with LLL for LBBPa can be diagnosed after a marked fall in the current of injury (COI) amplitude associated with a “QS” morphology, along with a drop in pacing impedance to <450 Ω. In the case of perforation, the lead can be advanced in the left ventricle and can evoke left ventricular ectopy. Pacing thresholds can rise.
- Macrodislodgment. Macroscopic dislodgment of the lead should be addressed as a technical problem during lead implantation (i.e., drill effect during screwing) and may lead to loss of capture. Lead displacement can be both in the right ventricle or left ventricle in the case of concomitant septal perforation.
- Microdislodgment. Per-operative microdislodgments are diagnosed in case of a change in morphology at surface 12 lead ECG, with a loss of terminal R’/r’ deflection in V1 and/or widening of the QRS.
- Lead rupture. Subtricuspidal friction of the lead may lead to lead rupture. The most common point of rupture is between the lead body and the ring [18,19]. LLL can be affected, too, and fractured leads in atypical locations can suggest that multiple attempts during initial lead implantation may be at a higher risk of lead fracture [20].
- Helix damage. Endocardium entanglement or deep septal penetration in thick myocardium can hamper tissue advancement, and counterclock rotations to retract the helix can be ineffective. Repositioning maneuvers are often necessary during LBBPa and can be a source of possible complications. In rare cases, helix elongation can precede helix rupture into the interventricular septum [13].
- Chest pain and ST elevation. Acute coronary events are rare occurrences during LBBPa implants (<1% of implants). They generally present with intraprocedural chest pain and possible ST elevation. Helix and lead retraction usually leads to symptom resolution. Coronary angiography is negative when performed in these cases. However, persistent ST elevation should also prompt suspicion of coronary vasospasm.

## 6. Conclusions

**Figure 7:**
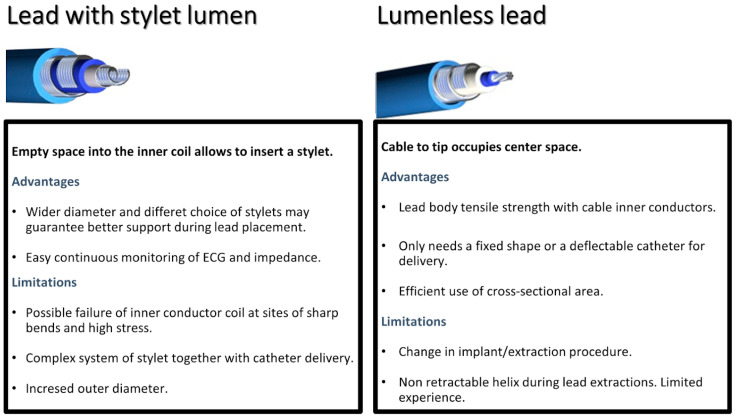
Comparison between SDL and LLL.

LBBPa has emerged as a promising technique for achieving left ventricular resynchronization through capture of the LBB. Compared with HBP, LBBPa offers several advantages, including lower thresholds, reduced displacement rates, and better resynchronization capabilities in cases of bundle branch block. The LLL, particularly the SelectSecure™ 3830 from Medtronic, has shown favorable outcomes in terms of stable pacing parameters and reduced complication rates. However, the advent of SDL, such as Solia S, Ingevity+, and Tendril, has provided additional options for LBBPa, demonstrating comparable success rates (Figure 7). Future advancements in LBBPa should focus on several key technical implementations: the introduction of more supportive delivery systems with radiopaque tips will enhance operator navigation and precision. There will be an increasing availability of devices specifically designed for physiological stimulation, ensuring more effective and targeted therapy. Additionally, the development of simplified screw fixation systems that allow for pacing during the insertion process will be crucial. These innovations will optimize the procedure, improve outcomes, and enhance wider adoption in clinical practice.

## Data Availability

No new data were created or analyzed in this study.
